# A novel neoantigen discovery approach based on chromatin high order conformation

**DOI:** 10.1186/s12920-020-0708-z

**Published:** 2020-08-27

**Authors:** Yi Shi, Mingxuan Zhang, Luming Meng, Xianbin Su, Xueying Shang, Zehua Guo, Qingjiao Li, Mengna Lin, Xin Zou, Qing Luo, Yaoliang Yu, Yanting Wu, Lintai Da, Tom Weidong Cai, Guang He, Ze-Guang Han

**Affiliations:** 1 Bio-X Institutes, Key Laboratory for the Genetics of Developmental and Neuropsychiatric Disorders, Shanghai Jiao Tong University, 1954 Huashan Road, Shanghai, 200030 China; 2 Shanghai Key Laboratory of Psychotic Disorders, and Brain Science and Technology Research Center, Shanghai Jiao Tong University, 1954 Huashan Road, Shanghai, 200030 China; 3 Key Laboratory of Systems Biomedicine, Ministry of Education. Shanghai Center for Systems Biomedicine, Shanghai Jiaotong University, Shanghai, 200240 China; 4 University of California at San Diego, 9500 Gilman Dr. La Jolla, San Diego, CA 92093 USA; 5 College of Biophotonics, South China Normal University, Guangzhou, 510631 China; 6 The Eighth Affiliated Hospital, Sun Yat-Sen University, Shenzhen, 518033 China; 7 University of California at Los Angeles, Los Angeles, CA 90095-1732 USA; 8 David R. Cheriton School of Computer Science, University of Waterloo, Waterloo, ON N2L 3G1 Canada; 9 International Peace Maternity and Child Health Hospital, Shanghai Jiaotong University School of Medicine, Shanghai, 200030 China; 10 School of Computer Science, The University of Sydney, Sydney, NSW 2006 Australia

**Keywords:** Neoantigen, 3D genome, Hi-C, Immunotherapy, pMHC, Epitope

## Abstract

**Background:**

High-throughput sequencing technology has yielded reliable and ultra-fast sequencing for DNA and RNA. For tumor cells of cancer patients, when combining the results of DNA and RNA sequencing, one can identify potential neoantigens that stimulate the immune response of the T cell. However, when the somatic mutations are abundant, it is computationally challenging to efficiently prioritize the identified neoantigen candidates according to their ability of activating the T cell immuno-response.

**Methods:**

Numerous prioritization or prediction approaches have been proposed to address this issue but none of them considers the original DNA loci of the neoantigens from the perspective of 3D genome. Based on our previous discoveries, we propose to investigate the distribution of neoantigens with different immunogenicity abilities in 3D genome and propose to adopt this important information into neoantigen prediction.

**Results:**

We retrospect the DNA origins of the immuno-positive and immuno-negative neoantigens in the context of 3D genome and discovered that DNA loci of the immuno-positive neoantigens and immuno-negative neoantigens have very different distribution pattern. Specifically, comparing to the background 3D genome, DNA loci of the immuno-positive neoantigens tend to locate at specific regions in the 3D genome. We thus used this information into neoantigen prediction and demonstrated the effectiveness of this approach.

**Conclusion:**

We believe that the 3D genome information will help to increase the precision of neoantigen prioritization and discovery and eventually benefit precision and personalized medicine in cancer immunotherapy.

## Background

In a variety of human malignancies, immunotherapies via boosting the endogenous T cell’s ability to destroying cancer cells have demonstrated therapeutic efficacy [1]. Based on clinical practices in a substantial fraction of patients, the inference of endogenous T cell with mounted cancer-killing ability is that the T cell receptor (TCR) is able to recognize peptide epitopes that are displayed on major histocompatibility complexes (MHCs) on the surface of the tumor cells. These cancer rejection epitopes may be derived from two origins: the first origin of potential cancer rejection antigens is formed by non-mutated proteins to which T cell tolerance is incomplete for instance, because of their restricted tissue expression pattern; the second origin of potential cancer rejection antigens is formed by peptides that cannot be found from the normal human genome, so-called neoantigens [1]. With the development of genome sequencing, it has been revealed that during cancer initiation and progression, tens to thousands of different somatic mutations are generated. Most of these mutations are passenger mutations, meaning there is no obvious growth advantage, and are often caused by genomic instability within the tumor cells. A limited number of cancer mutations are driver mutations which interfere with normal cell regulation and help to drive cancer growth and resistance to targeted therapies [2]. Both passenger mutations and driver mutations can be nonsynonymous that alter protein coding sequences, causing tumor to express abnormal proteins that cannot be found in normal cells. When cell metabolize, the proteins possessing abnormal sequences are cut into short peptides, namely epitopes, and are presented on the cell surface by the major histocompatibility complex (MHC, or human leukocyte antigen (HLA) in humans) which have a chance to be recognizable by T cell as foreign antigens [2]. Because each cancer patient has a unique somatic mutation combination, leading to personalized epitopes, the neoantigen therapy shed light on precision and personalized cancer immunotherapy.

According to the discoveries mentioned above, in theory therefore, if the potential neoantigens can be identified via sequencing technology, one can synthesize epitope peptides in vitro and validate their efficacy in vivo (cancer cell-line or in mouse model) before clinical practice [1, 2]. Indeed, cancers with a single dominant mutation can often be treated effectively by targeting the dominant driver mutation [2, 3]. However, when the somatic mutations are abundant, which is the case in most cancer types, it is computationally challenging to efficiently prioritize the identified neoantigen candidates according to their ability to activate the T cell’s immuno-response [4]. Over the past few decades, numerous neoantigen prediction approaches have been proposed to address this issue [5–7]. These approaches can be classified into two major categories: the protein 3D structure-based approaches, which consider the peptide-MHC (pMHC) and TCR 3D conformation, and the protein sequence-based approaches, which consider the amino acid sequence of protein antigens. For the protein 3D structure-based approaches, in some specific cases when high quality pMHC 3D structures are available, molecular dynamic (MD) methods are used to explore the contact affinity of pMHC-TCR complex [8–10], in most cases, however, the modelling or simulation by protein docking and threading has to be used due to the lack of high quality pMHC 3D structures. Most approaches belong to the sequence-based category as there are much larger data sets for training and validation [11, 12] and because they are usually very efficient to set up [4, 13].

Early sequence-based methods relied on position-specific scoring matrices (PSSMs), such as BIMAS [14] and SYFPEITHI [15], in which the PSSMs are defined from experimentally confirmed peptide binders of a particular MHC allele [4]. Later, more advanced methods based on machine-learning techniques have been developed to capture and utilize the nonlinear nature of the pMHC-TCR interaction which indeed demonstrated better performance than the PSSM-based methods. Consensus methods that combine multiple tools to obtain more reliable predictions were also developed, such as CONSENSUS [16] and NetMHCcons [17], which demonstrated better performances; for these methods however, the performance gain is determined by the weighting scheme which cost increased computational power. When considering peptide binding, most methods did not consider the HLA allele variety, therefore, pan-specific methods, such as NetMHCpan [6, 7], are developed which allow the HLA type independent prioritization.

As one of the widely adopted practices in neoantigen prioritization, NetMHCpan first trains a neural network based on multiple public datasets, then the affinity of a given peptide-MHC considering the polymorphic HLA types HLA-A, HLA-B or HLA-C is computed according to the trained neural network. NetMHCpan [7] and NetMHCIIpan [18] perform remarkably, even compared to allele-specific approaches [4, 19]. However, although several assessments and criteria were proposed in the past aiming at a more fair and effective comparison [19–21], there are no recent independent benchmark studies that can be used to recommend specific tools up until now. More importantly, however, to the best of our knowledge, none of the neoantigen prediction methods mentioned above consider the mutation DNA loci of the neoantigens in the perspective of 3D genome, which carries much richer information compared to the amino acid sequence alone [22]. In this work, we retrospect the DNA origin of the immuno-positive and immuno-negative neoantigens in the context of the 3D genome and demonstrate some discoveries that worth paying attention to.

## Methods

### Data collection and curation

All the peptide sequences and their corresponding immune effectiveness were collected from IEDB (T Cell Assay )[12] on May 27th 2018; the raw dataset contains 337,248 peptide records. We narrowed down to *Homo sapiens* and MHC-I subtypes and then further restrain the AA length to be equals to 9 with duplicated peptide merged. Finally, we obtained 3909 qualified records with 809 immuno-positive peptides and 3100 immuno-negative peptides that has mapping hits in the human hg19 reference genome. Note that for identical peptides with multiple immune experiments, we define positive rate > 0.8 as immuno-positive peptides and positive rate < 0.2 as immuno-negative peptides. In detail, there are two steps in this procedure: Step I. Extracting the *Homo sapiens* peptide sequences and cleaning up the dataset from initial dataset. We used the R package PANDAS to create a data frame object and assigned the column name by importing a name dictionary. Then we filtered the dataset so that the only entries left have “*Homo sapiens*” as their hostname and further cleaned up the dataset by filtering peptides with illegal amino acid alphabet. Step II: Counting the number of the sequences that have positive qualitative measure. We keep counting until the last appearance of a target sequence and increase the positive counter by one whenever a positive qualitative measure is detected. For the last appearance of the sequence, we can either add 1 if its positive or we skip to the next step. The counter resets every time we finish counting positivity for a sequence and move on to the next one. We store the counted values into a hash table where the sequence combining the MHC type serves as the hash key.

For the chromatin 3D conformation data, we used the Hi-C data of hESC and IMR90 cell lines generated by Bin Ren’s lab [23]. The contact frequencies and the subsequent chromatin 3D modeling are based on these Hi-C (genome-wise chromatin conformation capturing technology) data.

### Mapping peptides to human genome

To map the peptides to human genome (hg19), we wrote a pipeline to query the BLAST [24] web server and map the gene names to chromosomes and starting positions. The algorithm first divided the dataset into 711 folds where each fold has 100 sequences for the BLAST server to process. To set up the BLAST search, we regulated the searching algorithm to search for *Homo sapiens* matches only with entrez ID keywords and used the PAM30 matrix to search for matches. We also adjusted the gap costs to regulate gap penalty. After the setup, we called BLAST iteratively and wrote the result into a tsv file. For each match, we saved the accession and raw bit score for the first hit. After acquiring the accessions, we uploaded a list of refseq id to the DAVID tool [25] to obtain the gene names composed with gene symbols. The algorithm mapped gene names to chromosome positions, and we started with a dataset that records chromosome positions and gene names for numerous genes as our database. To save time during iterations, we created two dictionaries recording chromosome positions with gene names as keys, one from the dataset we produced from BLAST results and one from the database. We iterated through the dictionaries simultaneously. If we found a match for the keys, we recorded the chromosome positions in the result file. The final result is in the form of a tuple that contains peptides, HLA subtype, chromosome number, and chromosome position.

### Chromatin 3D modeling

We developed a new method for modeling 3D conformations of human genome using molecular dynamics (MD) based approach with resolution of 500 kb (bin size) for hESC and IMR90 Hi-C data. In this method, each bin was coarse-grained as one bead and intact genome was modeled as 23 polymer chains represented by bead-on-the-string structures. The spatial position of each bead is affected by two factors: (1) chromatin connectivity that constrains sequentially neighbor beads in close spatial proximity and (2) chromatin activity that ensures active regions are more likely to be located close to the center of cell nucleus. In this work, chromatin activity was estimated as compartment degree that can be directly calculated from Hi-C matrix with algorithm described in previous work [26]. Based on the relative values of compartment degrees, all the beads were assigned distances with different values to nuclear center and then the conformation of chromatin was optimized from random structures with molecular dynamics approach by applying bias potential to satisfy these distance constraints. For each cell linage, 300 conformation replicas were optimized from random ones to reduce possible bias for further analysis.

### Neoantigen prediction via 3D genome nearest neighboring

For a given target peptide, we first retrieve its 3D coordinates <x, y, z > based on the 3D modeling results mentioned above. For the training dataset, i.e., the peptides with known immunogenicity, we also retrieve their 3D coordinates. We then compute the Euclidean distances between the target peptide’s 3D genome coordinates with all the training peptides’ 3D coordinates. Note that we only consider those peptides whose corresponding chromosome are the same as the target peptide since intra-chromosomal distances are usually significantly closer than the inter-chromosomal distances due to the chromosome territory principle. We then collect the K-nearest neighbor (KNN) peptides and count the immunogenicity percentage, where k is chosen to be 10. The majority voting scheme is adopted to calculate the target peptides’ KNN prediction scores. The predicted scores were further combined with the state-of-the-art neoantigen prediction algorithm, i.e., netMHCpan to generate the final prediction scores. In detail, a netMHCPan prediction score is subtracted by the KNN prediction score to achieve the final immunogenicity prediction score. We term this method 3DGenome-NN. The neoantigen prediction is then the prioritization based on this score.

## Results

### Neoantigen proximity in individual chromosome (intra-chromosome)

**Fig. 1:**
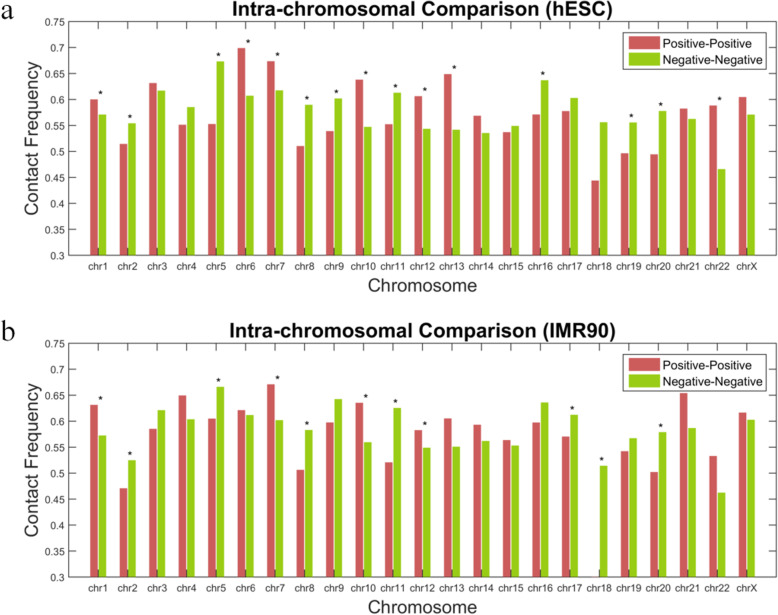
Average contact frequency (CF) of immuno-positive peptide pairs and immuno-negative peptide pairs based on **a** hESC Hi-C data and **b** IMR90 Hi-C data. The star sign indicating *p* < 0.05

We generated all peptide pairs between immuno-positive peptides and peptide pairs between immuno-negative peptides. Then on each chromosome (intra-chromosome), we generate each pair’s contact frequency on hESC and IMR90 Hi-C data [23]. The results are shown in Fig. 1a and b. Jointly from these results, we found that positive peptides’ corresponding DNA loci tend to be more proximate (*p* < 0.05) than the negative ones on chromosome 1 (chr1), chr7, chr10, and chr12, while negative peptides’ corresponding DNA loci tend to be more proximate than the positive ones on chromosome chr2, chr5, chr8, chr11, and chr20.

### Neoantigen proximity in the whole genome (inter-chromosome)

**Fig. 2:**
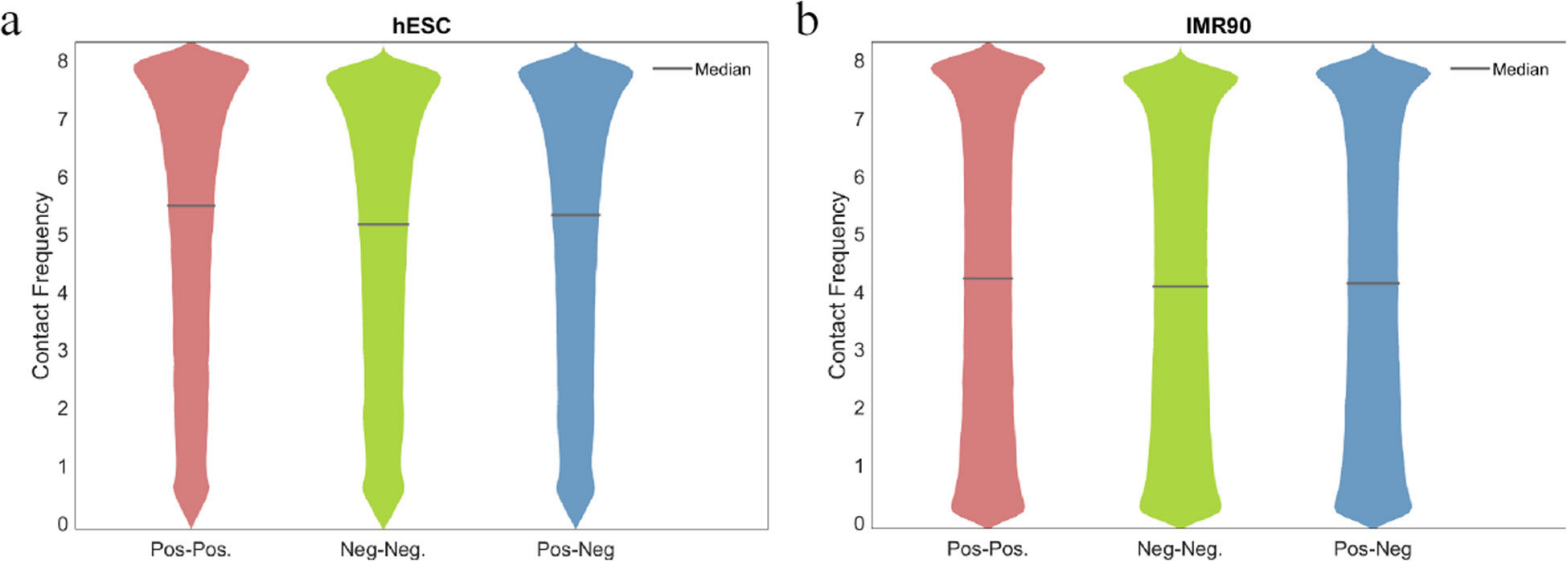
Contact frequency distribution comparison between immuno-positive peptide pair’s DNA loci, immuno-negative peptide pairs’ DNA loci, and immuno-positive-negative peptide pairs’ DNA loci, on **a** hESC Hi-C data and **b** IM4R90 Hi-C data

For the inter-chromosomal peptide pairs, i.e., three types of immuno-pos.-pos. Pairs, immuno-neg.-neg. Pairs, and immune-pos.-neg. Pairs, we collect their contact frequencies based on the hESC and IMR90 Hi-C datasets respectively, and calculate the average values and median values. Figure 2 demonstrates the distributions of the contact frequencies of all the three types of peptide pairs on the two Hi-C datasets respectively. We found that the immuno-pos.-pos. Peptide pairs are statistically more proximate to each other comparing to immuno-neg.-neg. Peptide pairs, while the immune-pos.-neg. Pairs’ proximities are in between; the corresponding *P*-values are all smaller than 1 × 10^− 99^ (note: large sample size, i.e., number of pairs, also contribute to small *P*-values).

Combining the results shown in Figs. 1 and 2, it tells us that globally (inter-chr.), immuno-positive peptide pairs tend to cluster more than the immuno-negative peptide pairs, while locally (intra-chr.), it varies from chromosome to chromosome, and in general, this piece of information contributes to prioritizing peptide’s immunogenicity.

### Neoantigen prediction results

**Fig. 3:**
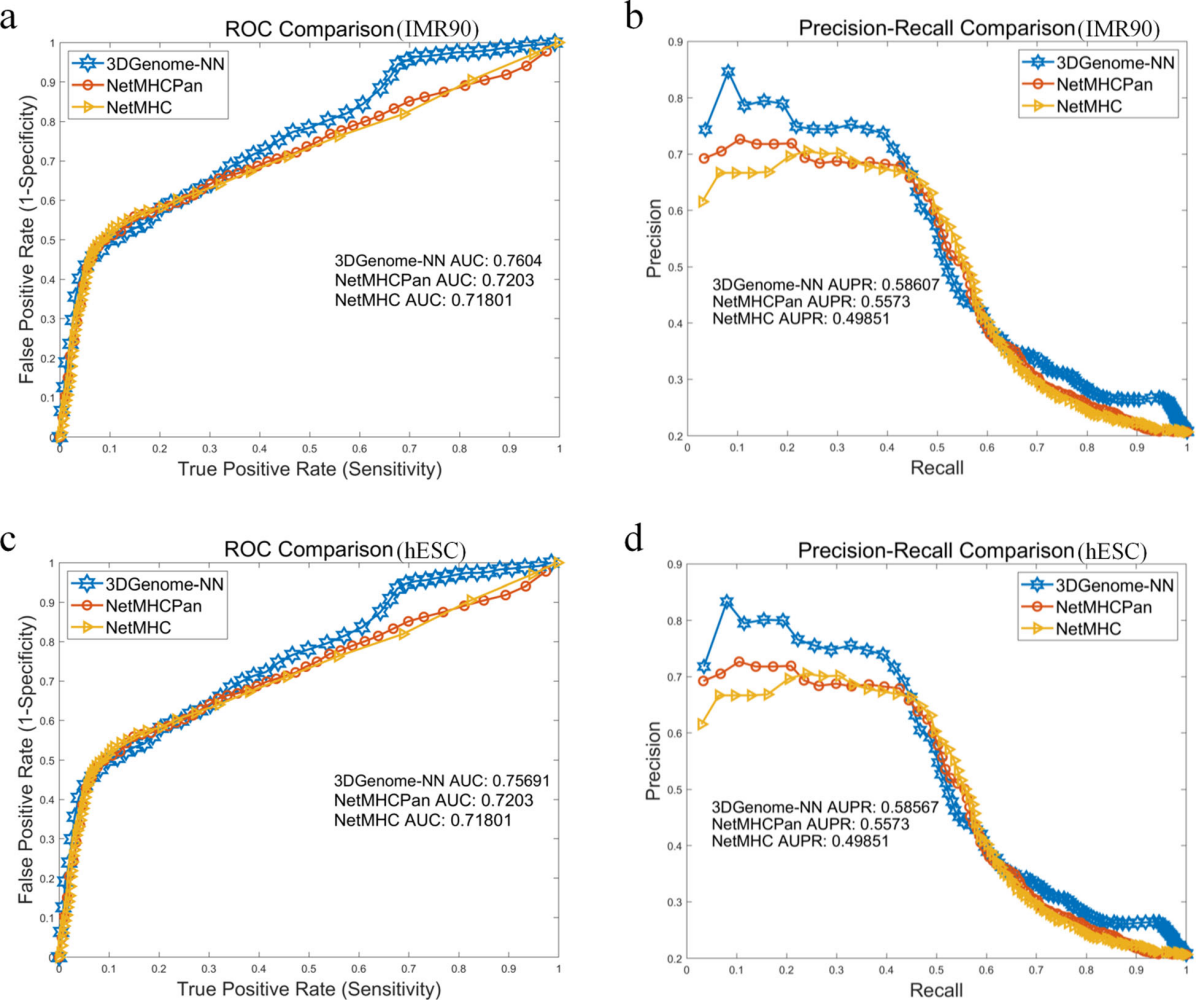
The prediction effectiveness of the 3D Genome Nearest Neighboring algorithm (3DGenome-NN) comparing to the state-of-the-art algorithms NetMHC and NetMHCpan. **a** and **c** demonstrate the ROC curve of 3DGenome-NN, NetMHC, and NetMHCpan respectively. **b** and **d** demonstrate the Precission-Recall curve of 3DGenome-NN, NetMHC, and NetMHCpan respectively

We adopted the leave-one-out cross validation scheme to compare the neoantigen prediction effectiveness between our proposed method 3D Genome Nearest Neighboring (3DGenome-NN) described in subsection 2.4 of the Methods section and the current state-of-the-art algorithms NetMHC and NetMHCpan. After prediction for each target peptide, we obtain a prediction score vector, we then collected their corresponding known immunogenicity vector (ground truth) and calculate and plotted their ROC curve and Precision-Recall curve as shown in Fig. 3. Based on the AUC (area under ROC) and AUPR (area under precision-recall) scores, we demonstrate that 3DGenome-NN outperforms NetMHC and NetMHCpan to a significant level in distinguishing immuno-positive neoantigens and immuno-negative neoantigens. As this gain of discriminative power is due to the employment of 3D genome information, it supports the conjecture that the distributions of the DNA origins of the immuno-positive peptides and immuno-negative peptides are not random on the 3D genome but obey certain patterns.

## Discussion

In cancer immune therapy, neoantigen therapy is a rising and promising topic as it can be genuinely personalized and precise. However, when the somatic mutations are abundant, it is computationally hard to efficiently prioritize the identified neoantigen candidates according to their ability of activating the T cell immuno-response and numerous prioritization or prediction approaches have been proposed to address this issue. However, none of the existing approaches considers the original DNA loci of the neoantigens in the 3D genome perspective, to the best of our knowledge. In this work, we retrospect the DNA origin of the immuno-positive and immuno-negative neoantigens in the context of 3D genome and discovered that immuno-positive and immuno-negative neoantigens’ corresponding DNA tend to cluster differently in different chromosomes (intra-chromosome) and tend to cluster genome-wise (inter-chromosome). Specifically, immuno-active neoantigens’ corresponding DNA tend to locate at specific regions in the 3D genome. We therefore believe that by adopting the 3D genome information in advanced machine learning [27–29] and feature selection technologies [30–32], more precise neoantigen prioritization and discovery can be achieved and may eventually benefit precision medicine in cancer immunotherapy.

## Conclusion

In this work, we discovered that the corresponding DNA loci of the immuno-positive and immuno-negative neoantigens are distributed differently in the 3D genome space. Specifically, in some chromosomes, positive ones tend to cluster together, comparing to negative ones. In whole-genome scale, this also holds true. We discovered that by incorporating the 3D genome information into existing neoantigen prediction methods, better prediction accuracies can be achieved. We thus believe that the 3D genome information can increase the precision of neoantigen prioritization and discovery and eventually benefit precision and personalized medicine in cancer immunotherapy.

## Data Availability

The datasets used and/or analyzed during the current study are available from the corresponding author on reasonable request.

## Abbreviations

TCR: T Cell Receptor
MHC: Major Histocompatibility Complex
pMHC-I: peptide-MHC Class I
HLA: Human Leukocyte Antigen
